# The biochemical mechanism of hypoxia-induced mobilization of glycogen in cultured cancer cell

**DOI:** 10.1186/2049-3002-2-S1-P37

**Published:** 2014-05-28

**Authors:** Mung Kwan Long, Wong Nai Sum

**Affiliations:** 1 Department of Biochemistry, The University of Hong Kong, Hong Kong, Hong Kong

## Background

Metabolic reprogramming is one of the strategies adopted by cancer cells to survive hypoxic conditions. Recent findings suggest that hypoxic cancer cells derive the energy that they need through glycolysis using glucose mobilized from intracellular glycogen reserve. Glycogen phosphorylase (GP) is the major rate-determining enzyme for glycogen mobilization in many normal cells under the condition of starvation or physical exercise. The lysosomal alpha-glucosidase (GAA) has also been implicated in glycogen mobilization since deficiency of this enzyme is known to result in Glycogen Storage Disease Type II (Pompe disease). Although a role for GP in the mobilization of glycogen by cancer cells depleted of oxygen had been demonstrated, whether a similar role for GP (and GAA) could be demonstrated under deprivation of both glucose and oxygen is not yet known. This issue is addressed in this study by examining the intracellular level of glycogen in the absence and presence of pharmacological inhibitors of GP and/or GAA.

## Materials and methods

The Hela, HEK293 and HT29 cancer cell lines were subjected to hypoxic conditions (1%O_2_) in the presence or absence of glucose for 3 hours. The effect of these hypoxic conditions on intracellular glycogen level was examined in the presence and absence of inhibitors for glycogen phosphorylase [CAS 648926-15-2] or lysosomal alpha glucosidase (castanospermine or miglitol).

## Results

We showed that steady-state intracellular glycogen is significantly lowered (>50%) only when cultured cancer cell (Hela, HEK293 and HT29) were deprived of both glucose and oxygen for 3 hours. The hypoxia-induced lowering of intracellular glycogen was alleviated by the presence of glycogen phosphorylase inhibitor (CAS 648926-15-2) but not alpha glucosidase inhibitor (castanospermine or miglitol). The simultaneous presence of inhibitors for both enzymes did not have a synergistic effect in preventing hypoxia-induced glycogen mobilization as compared to that observed for (CAS 648926-15-2) alone. Hypoxia had no significant effect on both *in vitro* glycogen phosphorylase activity and mRNA level of glycogen phosphorylase. The total abundance of glycogen synthase and its phosphorylated form remained unchanged upon hypoxic treatment, suggesting that down-regulation of glycogen synthesis is unlikely to contribute to glycogen depletion. Addition of cycloheximide and actinomycin D had no effect on hypoxia-induced mobilization of glycogen, suggesting that *de novo* protein synthesis and mRNA synthesis are not required for hypoxia-induced glycogen consumption. These data suggest that glycogen phosphorylase plays a dominant role in glycogen utilization under hypoxic conditions in the absence of extracellular glucose.

